# Integrated value-chain and risk assessment of Pig-Related Zoonoses in Ghana

**DOI:** 10.1371/journal.pone.0224918

**Published:** 2019-11-11

**Authors:** Ayodele O. Majekodunmi, Henry Ofosu Addo, Husein Bagulo, Langbong Bimi

**Affiliations:** 1 Livestock and Poultry Research Centre, College of Basic and Applied Sciences, University of Ghana, Legon, Accra, Ghana; 2 Department of Animal Biology & Conservation Science, College of Basic and Applied Sciences, University of Ghana, Legon, Accra, Ghana; Kent State University, UNITED STATES

## Abstract

The marked increase in the pig-trade in Ghana has raised concerns about increased transmission of related zoonotic diseases. A study on pig-related zoonoses along the pork value-chain was conducted in Greater Accra and Upper East Regions of Ghana. Results showed significant taenia (60%) and trichinella (8%) seroprevalence in pigs in Upper East with little evidence of transmission to humans. Sero-prevalence of HEV was high in both pigs (85%) and humans (37%). Sero-prevalence rates were significantly higher in Upper East than Greater Accra. Pig handlers in Accra had significantly higher sero-prevalence rates (58%) than other community members (18%) but there was no such association in the Upper East. Given the high rates of mortality, miscarriage and stillbirth associated with HEV in pregnancy, it is a cause for concern that 31% women of child-bearing age tested sero-positive for HEV.

## Introduction

Global livestock production has increased steadily over the past three decades with associated increases in risk of zoonotic disease [1, 2]. Increases have been recorded particularly in the poultry and pork sectors in low and middle income countries. Pig production in Ghana has increased at a rate of 10.5% annually over the last 15 years, both in terms of intensive/commercial and extensive/free-ranging animals. However demand still exceeds domestic production by 20% [3, 4].

There is concern that the rapid increase in smallholder pig production across Ghana may exacerbate the risk to human health of pig-associated zoonoses such as *Taenia solium*, *Trichinella spiralis* and hepatitis E virus. The transmission of these diseases is strongly linked to poor sanitation and health and safety practices in meat processing [5, 6]. Ghana has a particularly poor sanitation record, with just 19% sanitation coverage and high open defaecation rates [7]. There is also evidence of widespread poor practices throughout livestock value-chains and corresponding zoonotic and food-borne disease in at-risk populations [8–14]

With the increase in pig consumption and production set to continue, it is important to improve our current knowledge of pig-associated zoonosis burdens in Ghana and other countries. This paper presents results of a study investigating taenia, trichinella and HEV along the pork value-chain in Ghana.

## Methods

### Study area

**Fig 1:**
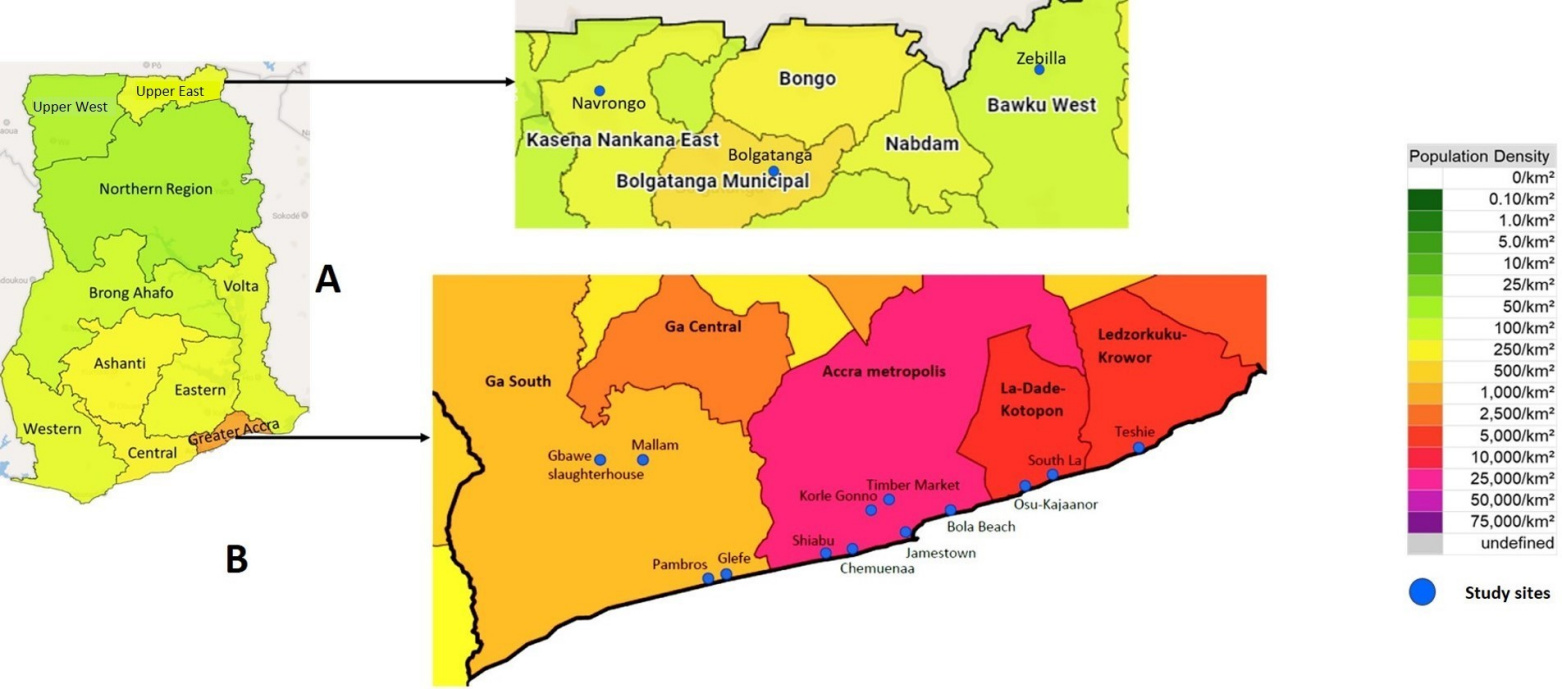
Study sites. (A) Upper East Region (B)Greater Accra Region.

This study was conducted in 10 study sites across four coastal municipalities in the Greater Accra Region of Ghana (Fig 1), original settlements of the Ga people which grew into the present day capital city of Accra. These areas are characterized by high population and poverty rates, low rates of education and sanitation, poor provision of public amenities, poor access to sanitation facilities and potable water. They are enclaves of a more traditional Ga lifestyle within the city of Accra, where traditional authorities have a lot of influence. Fishing and livestock, particularly pigs, are important sources of income Pigs are housed at night in makeshift wooden pens, often on the beach, and allowed to roam during the day. A few intensive systems are present, with pigs confined in concrete pens. The study sites in Greater Accra include 1 dedicated pig slaughterhouse receiving large numbers of pigs from the Upper East Region.

Three locations in the Upper East Region (Zebilla, Navrongo, Bolgatanga), identified as major supply areas for pigs to Accra were also included in the study. Additional pigs from across the Upper East Region were sampled at the point of sale after aggregation and transport to Accra. In the Upper East, the dominant production system is mixed cropping with extensive livestock production. Most households own a few chickens, pigs and/or small ruminants. Pigs are allowed to roam free and scavenge in the dry season. Once fields are planted at the beginning of the wet season, pigs are sold off and breeding stock are confined until after harvest to prevent crop destruction.

### Study design

Study sites were chosen purposively as they represent the main locations for pig production and trade in the Upper East region and along the coast of Greater Accra. A value-chain mapping exercise was conducted between November–December 2017 targeting pig farmers, butchers and pork retailers. Individual structured questionnaires were administered to pork retailers and focus group discussions were conducted with pig farmers and butchers. Both activities focused on collecting data on the movement of pigs and pork along the value-chain; knowledge of zoonoses and food safety; and interactions with environmental, veterinary and public health officials.

Between January–July 2018 a serological survey was conducted, collecting blood samples from pigs, pig farmers, butchers, pork retailers, and community members in households adjacent to pig farms. These groups were considered at-risk individuals due to their direct contact with pig and pork products and close proximity to pig farms.

### Sample size and selection

Human sample size was determined based on the hypothesis that prevalence in pig/pork handlers is higher than in groups with no direct contact with pigs. At 50% seroprevalence in pig-handlers, 5% background sero-prevalence in the rest of the population, 80% power and 5% precision, the minimum sample size in each group was determined to be 19. A purposive sampling frame was employed to target actors within pork value chain. Subjects were recruited through engagement with local associations of pig-farmers and pork retailers. Pre-survey activities identified a total of 100 farmers, 25 butchers, and 94 pork sellers and 1,000 pigs in the Greater Accra survey area. In the Upper East, 62 farmers, 24 butchers, and 7 pork sellers were identified (Table 1). All households in close proximity to pig farms were approached to recruit community members with no occupational exposure to pigs. All consenting participants were enrolled in the study. A final sample size of 238 humans was achieved in Greater Accra and 84 in Upper East (Table 2).

**Table 1:** Sampling frame.

Municipality	Population*	Study site	No. Farmers	No. butchers	No. Pork Retailers	confined	Free-range
Ga South	521,162	Gbawe farm	1	25	9	30	
		Mallam	0	0	11	0	0
		Pambros	2	0	0	30	22
		Glefe	2	0	0	38	
		Other	0	0	13	0	0
Accra Metropolitan	2,087,668	Shiabu Beach	6	0	0	0	100
		Korle Gonno	45	0	0	400	0
		Chemuenaa	9	0	0	0	54
		Jamestown	0	0	9	0	0
		Bola Beach	1	0	0	0	20
		Pig Farm	0	0	31	0	0
		Timber Market	5	0	0	0	32
		Osu	0	0	13	0	0
		Other	0		16	0	0
La-Dade-Kotopon	221,284	Osu-Kajaanor	15	0	0	0	200
		South La	9	0	1		180
Ledzorkuku-Krowor	275,239	Teshie	5	0	0	30	35
**Total**	**3,105,353**		**100**	**25**	**94**	**528**	**589**
Kasena Nankana East	133,610	Navrongo	15	3	5	0	85
Bolgatanga Municipal	160,308	Bolgatanga	24	6	0	0	145
Bawku West	114,526	Zebilla	23	15	2	0	58
**Total**	**388,444**		**62**	**24**	**7**	**0**	**188**

*Ghana Statistical Service, 2010

**Table 2:** Study participants.

Category	Greater Accra				Upper East			
	No.	Mean Age	♀	♂	No.	Mean Age	♀	♂
Farmers	99	42.5	3	96	36	43.7	14	22
Community Members	67	35.2	22	45	36	38.9	9	27
Butchers	20	36.2	3	17	6	36.5	0	6
Pork Sellers	52	41.3	50	2	6	31.7	1	5
**Total**	**238**	**39.6**	**80**	**158**	**84**	**40.1**	**24**	**60**

The minimum sample size required to determine seroprevalence in pigs using a simple random sample at 50% prevalence, 80% power and 95% precision was 280. Pigs were randomly selected for sampling at each site using probability proportional to population. However, only 247 pigs were sampled, 102 in Greater Accra and 145 in Upper East due to problems with owner consent.

### Serological methods

Blood (10 ml) samples from all human participants and pigs were collected in plain vacutainers, labelled and transported on ice to the National Public Health Reference Laboratory, Korle Bu. The samples were then centrifuged and sera stored at -81°C for serological analysis.

Sera from humans were tested for the presence of antibodies against HEV (IgM, IgG, IgA) using MP Diagnostic HEV ELISA 4.0 kits (sensitivity 98%; specificity 97% MP Diagnostics, Singapore); antibodies against Taenia solium (IgG) using Novatec *Taenia solium* (sensitivity 94%; specificity 95%; Novatec Immundiagnostica, Germany);and antibodies against Trichinella Spiralis (IgG) using *T*. *Spiralis* ELISA IBL (sensitivity 100%; specificity 95%; IBL International, Germany).

Sera from pigs were tested for the presence of antibodies against HEV (IgM, IgG, IgA) using MP Diagnostic HEV ELISA 4.0 Vet (sensitivity 98%; specificity 97%; MP Diagnostics, Singapore), antibodies against Trichinella Spiralis (IgG) using Priocheck Porcine Trichinella Ab 450 (sensitivity 97%; specificity 98%; Prionincs, Switzerland) and antibodies against Taenia solium taeniosis (IgG) using Novatec Vetline *Taenia solium* (sensitivity 95%; specificity 95%; Novatec Immundiagnostica, Germany).

### Value-chain assessment

Key informant interviews were conducted with selected members of District departments of Environment, Agriculture and Urban Planning, to understand the policy and regulatory environment in which the pig trade operates. Focus group discussions were conducted with value-chain actors to understand the motivations and constraints in smallholder pig production and trade; and to track pig movements along the value-chain in Ghana (Table 3). Data was extracted into MS Excel sheets and analysed along the themes of locations, business performance, linkages. Water, sanitation and hygiene (WASH) and knowledge of zoonotic diseases were also explored.

**Table 3:** Value chain focus group discussions.

Category	Location	No. Participants*	Mean Age	Years of schooling	♀	♂
Farmers	Gbawe	15	36.7	7.6	0	15
Butchers	Gbawe	9	32.1	8.8	0	9
Farmers	Chemuenaa	7	39.3	8.7	0	7
Farmers	Shiabu, Glefe & Pambros	21	38.2	6.2	2	19
Farmers	Bolgatanga	12	27.7	8.9	0	12
Farmers	Korle Gonno	27	47.3	10.1	2	25
Farmers	Teshie	12	42.2	8.3	1	11
Farmers	Zebilla	7	31.3	9.3	0	7
Pork Retailers	Accra	42	40.5	10.3	44	7
Consumers	Accra	7	43	9.3	0	7

*Maximum FGD size was 12 participants. Where more participants were present, they were split into smaller groups.

### Data analysis

Questionnaire and laboratory data were analysed using SPSS software (IBM, USA version 23, New York, NY, USA). Multiple logistic regression was performed to determine significant associations between serological results and variables such as level of contact with pigs and production system (Table 4). The conventional asymptotic significance (p) level of 0.05 was subsequently used to determine the level of significance between the variables, with odds ratios (OR) used to estimate the risk within a 95% confidence interval.

**Table 4:** Logistic regression variables.

Variable	Type of Variable	Class of Variable
Hepatitis E/Taenia/Trichinella infection	Outcome variable	Binomial positive = 1, negative = 0
Occupation	Predictor variable	Ordinal Farmer = 4 Butcher = 3 Pork Seller = 2 Community member = 1
Location	Predictor variable	Categorical
Sex	Predictor variable	Binomial Male = 1, Female = 0
Age	Predictor variable	Continuous
Production System	Predictor Variable	Binomial Free-range = 1; Confined = 0

### Ethical statement

Ethical approval for this study was granted by the Ethics Committee for Basic and Applied Sciences (ECBAS) of the College of Basic and Applied Sciences, University of Ghana (Application No. ECBAS010/17-18). Written consent was acquired from all participants and owners of pigs sampled.

## Results

### Value-chain mapping

**Fig 2:**
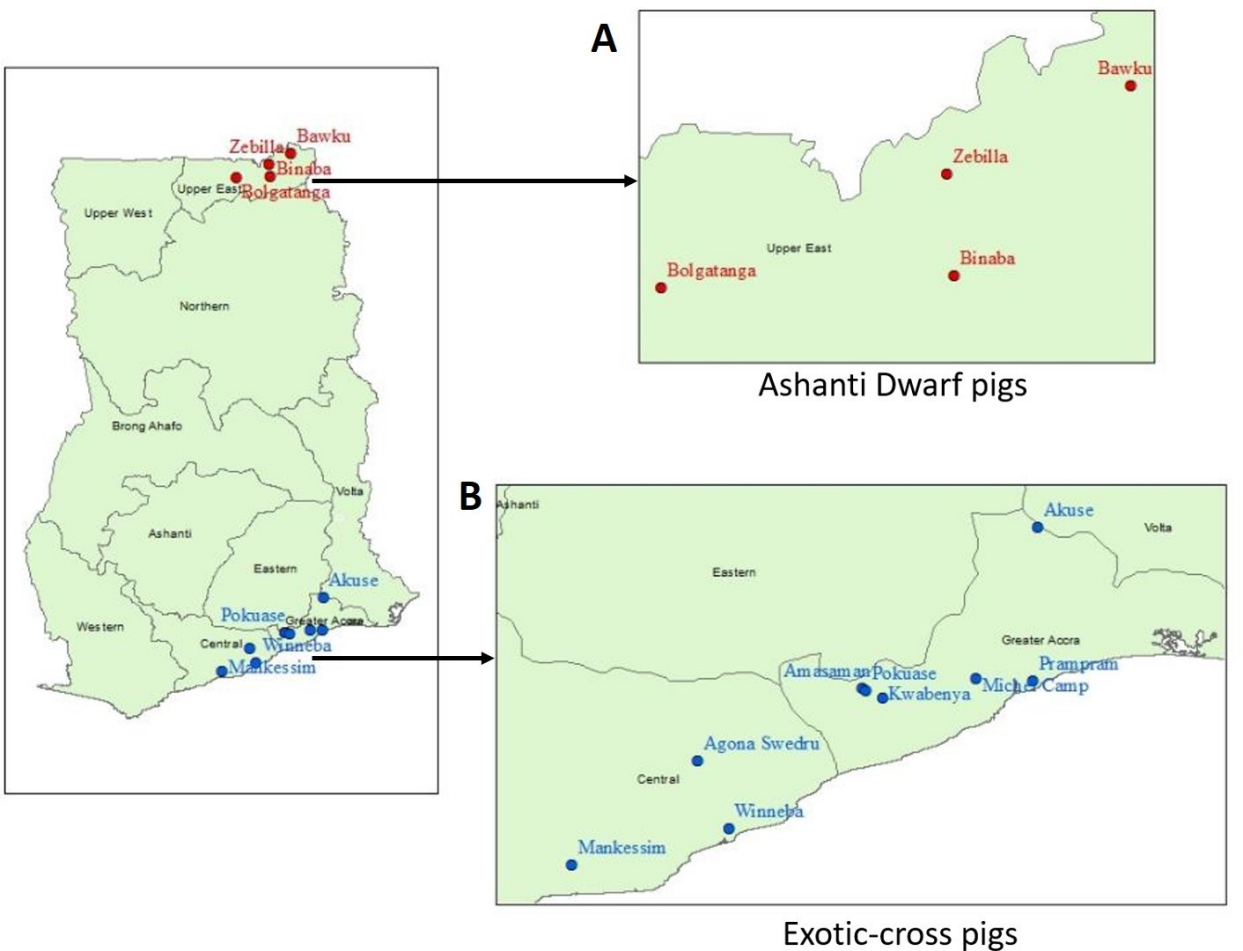
Live pig supply sites to Accra. (A) Ashanti Dwarf pigs (B) Exotic-cross pigs.

Demand for pork in Ghana is high, making pig-farming a profitable business. Many farmers and livestock traders who previously dealt in small ruminants have switched to pigs in recent years as the demand and profits are higher and incidents of theft are considerably lower. Two parallel supply chains for live pigs in Accra were identified (Fig 2).

Ashanti dwarf pigs raised on extensive and semi-intensive mixed farms in northern Ghana are sold at village markets to Accra-based pig traders. These traders aggregate the pigs in large towns such as Bolgatanga where they are inspected by the veterinary services. Pigs destined for human consumption must be checked for sub-lingual cysts as part of routine veterinary preventive health procedures. A transportation permit is also required to transport trade animals between districts. They are then transported to Accra by truck in consignments of 80–120 pigs. In Accra pigs are slaughtered, dressed and sold to pork retailers (and the occasional consumer). A few are sold live to local pig farmers as breeding or fattening stock. Pigs are available from Northern Ghana only in the dry season between December and June. In the wet season pigs cannot scavenge due to the risk of crop-damage and farmers find it hard to feed the confined pigs.

Smaller numbers of exotic and exotic-cross pigs are raised in intensive and semi-intensive commercial or government farms in southern Ghana (Accra, eastern and central regions) and sold to Accra-based pig traders. They also undergo veterinary inspection before transport by truck to Accra. The majority of these pigs are sold to local farmers as breeding stock and supplied to supermarkets. The rest are slaughtered and dressed for sale to pork retailers and consumers. Pigs are available all year round from this system.

Gbawe is a dedicated pig slaughter house. There are 2 slaughter slab operators, each with facilities for housing pigs. Most of the pigs available for sale here come from Northern Ghana. It is one of the few sites in Accra where customers can select their own pigs for slaughter. It serves private consumers, small-scale retailers who buy a single pig every week and larger concerns that buy up to 5 pigs per week.

### Policies and practices along the value-chain–food safety and zoonoses transmission

#### Farmer practices

Pig keeping in residential areas is illegal according to the by-laws of the Greater Accra region, unlike poultry and small ruminants. Therefore, apart from enforcing a total ban (which is not feasible) there is no legislative framework to regulate pig production in these areas. As such there is minimal coverage by veterinary or public health services. Environmental services do have some interaction with the pig-farmers, mostly focused on bad smells. Public planning departments focus on urban agriculture is mostly focused on vegetable growers, with little awareness and no provision for urban livestock enterprises.

Pigs roam free and are often found at refuse dumps and open defaecation sites or wallowing in surface water including canals draining into the sea. Free-range pigs are often deliberately kept close to open defaecation sites, regarded as a good food source. Waste from pig pens is often dumped directly into the sea. Pigs have been known to attack and kill young children. Free range farmers tend to have more pigs (25–300), while those with confined pigs have between 15–50.

About 30% of farmers provide slaughter and dressing services for their customers. There is a general awareness amongst farmers that cysts in carcasses are 'unhealthy' and farmers accept the loss when a customer rejects such a carcass. However, only 1 person interviewed (a retired teacher) understood the origin of cysts and their relationship to tapeworms or sanitation.

#### Butcher practices–Gbawe slaughterhouse

On average, fifty pigs are slaughtered at Gbawe Slaughterhouse every week. No planning provision is made for dedicated pig slaughter facilities by urban planning authorities. For religious reasons, these must be kept separate from facilities where other livestock are slaughtered. Therefore, pig slaughter is perforce informal. The facilities at Gbawe lack proper infrastructure and personal protective equipment for staff. There are low standards of sanitation and hygiene. There is no pipe-borne water or proper sanitation facilities. Instead, water is sourced from an on-site well. Waste from pig pens and carcasses are disposed of at an open dumping site close by and there is significant presence of scavenging dogs and carrion birds. Disinfectants are not used; in any case pigs are slaughtered and dressed on concrete flooring which, being porous, cannot be thoroughly disinfected.

The only interaction with authorities is with the environmental services who inspect regularly and give some training on sanitation. There is no meat inspection by veterinary services. Butchers are aware that cysts are ‘unhealthy’ and will not sell carcasses with cysts. The customer is advised to pick another pig and the seller bears the cost. However, there is little understanding of the origin of cysts or of their relationship to tapeworms or sanitation.

#### Pork-retailer practices

Retailers select animals that appear healthy, with a thin layer of fat. Once slaughtered, carcasses are cut into ~2kg pieces. Offal is dressed and packaged separately. They are aware that cysts are unhealthy and reject such carcasses. Roughly 20% of retailers steam these large pieces of pork at Gbawe before taking them home–often in the same vessels used for scalding pig carcasses. Pork retailers sell cooked pork (boiled, fried, grilled or stewed) 50% at bars and 50% at food stalls. Food selling premises are regularly inspected by public health and environmental services. They must undergo annual blood and stool tests for food-borne diseases such as E. coli and cholera. They have a good awareness of food-borne diseases and food safety practices but no awareness of zoonotic disease transmission.

### Serology

Of the 238 people tested in the Greater Accra region, 73 (31%) were sero-positive for HEV and 10 (4%) were sero-positive for T. solium as shown in Table 5. No persons sero-positive for Trichinella were detected. Pig handlers had a significantly higher HEV sero-prevalence (37%) than other community members (15%; z = 3.298; p = 0.001). Pig farmers had the highest prevalence at 49%, followed by pork retailers (21%) and butchers (20%). Community members with no occupational exposure to pigs/pork had the lowest prevalence at 15%.

**Table 5:** Seroprevalence of pig-related zoonoses.

Humans		HEV		Taenia		Trichinella	N
**Accra**							
**Farmers**	48	48%	3	3%	0	0%	99
**Butchers**	4	20%	1	5%	0	0%	20
**Pork sellers**	11	21%	0	0%	0	0%	52
**Community**	6	15%	6	9%	0	0%	67
**Total**	73	31%	10	4%	0	0%	238
**Upper East**							
**Farmers**	21	58%	1	3%	0	0%	36
**Butchers**	3	50%	0	0%	0	0%	6
**Pork Retailers**	2	33%	0	0%	0	0%	6
**Community**	21	58%	1	3%	0	0%	36
**Total**	47	56%	2	2%	0	0%	84
**Pigs**		**HEV**		**Taenia**		**Trichinella**	**N**
**Accra**							
**Confined**	9	41%		0%	0	0%	0
**Free-range**	73	91%		0%	0	0%	1
**Total**	**82**	**80%**			0		**102**
**Upper East**							
**Free-range**	126	88%	86	60%	11	8%	143

Logistic regression confirmed that HEV sero-prevalence in humans was significantly associated with occupational exposure to pigs/pork (Table 6). Each group ascending the value chain from consumers/community members to farmers was 1.5 times more likely to be infected with HEV (Odds ratio 1.4594; p = 0.0288) Men were 2.4 times more likely to be infected (Odds ratio 2.4474; p = 0.0092) with a prevalence of 36.7% compared to 18.8% in women.

**Table 6:** Logistic regression analysis.

**Human HEV Accra**	**165 cases have Y = 0; 73 cases have Y = 1**					
	**Chi Square = 17.9840; df = 4; p = 0.0030**					
Variable	Coeff.	StdErr	p	O.R.	Low --	High
Location	-0.0094	0.0385	0.8231	0.9906	0.9185	1.0684
Occupation	-0.378	0.1744	0.0288	1.4594	1.0087	2.0542
Sex	-0.895	0.3493	0.0092	2.4474	1.2343	4.8520
Age	0.0251	0.0138	0.0681	1.0254	0.998	1.0536
Intercept	-0.5268	0.7584	0.4475			
**Pig HEV Accra**	**21 cases have Y = 0; 81 cases have Y = 1**					
	**Chi Square = 28.1524; df = 2; p = 0.0000**					
**Variable**	**Coeff.**	**StdErr**	**p**	**O.R.**	**Low --**	**High**
Location	0.0576	0.0684	0.3996	1.0593	0.9264	1.2114
Production system	2.9508	0.6056	0.0000	19.1205	5.8348	62.6574
Intercept	-0.9364	0.6350	0.1403			
**Human Taenia Accra**	**228 cases have Y = 0; 10 cases have Y = 1**					
	**Chi Square = 7.1242; df = 4; p = 0.0284**					
Variable	Coeff.	StdErr	p	O.R.	Low --	High
Location	-0.2656	0.1265	0.0358	1.3041	0.9825	1.6711
Occupation	-0.121	0.4667	0.7954	0.886	0.355	2.2115
Sex	0.0915	0.719	0.8988	0.9126	0.223	3.7349
Age	0.0008	0.0301	0.9795	0.9992	0.9419	1.06
Intercept	-1.7104	1.2949	0.1865			
**Human HEV Upper East**	**Chi Square = 5.9663; df = 4; p = 0.2017**					
	**37 cases have Y = 0; 47 cases have Y = 1**					
Variable	Coeff.	StdErr	p	O.R.	Low --	High
Location	-0.4059	0.3295	0.2179	0.6663	0.3493	1.2711
Occupation	-0.0232	0.1725	0.8928	0.977	0.6967	1.3701
Sex	0.9063	0.7181	0.2069	2.4751	0.6058	10.1123
Age	0.0161	0.0171	0.3482	1.0162	0.9826	1.0509
Intercept	0.2372	1.1021	0.8296			

Logistic regression also showed that taenia sero-prevalence was significantly associated with location, with all taenia cases clustered in 4 out of 13 locations (Bola Beach, Gbawe, Chemuenaa & Kajaanor). Three persons were sero-positive for both taenia and HEV–two farmers from Kajaanor and one community member from Chemuenaa.

Of the 84 people tested in the Upper East region, 47 (56%) were sero-positive for HEV and 2 (2%) were seropositive for Taenia. No persons sero-positive for Trichinella were detected. Neither HEV nor Taenia sero-prevalence was significantly associated with age, sex, location or occupational exposure to pigs or pork.

Overall sero-prevalence of human HEV was 37%. Women of childbearing age (13–50 years) had 25% HEV sero-prevalence (67% in Upper East, 26% in Accra). HEV sero-prevalence was significantly higher in the upper east than in greater Accra (z = 4.12; p = 0.001). There was no significant difference btw taenia sero-prevalence in the two study areas (fishers exact test p = 0.7381).

Of the 102 pigs tested in the Greater Accra region, 82 (80%) were sero-positive for HEV. No pigs sero-positive for taenia or trichinella were detected. Logistic regression showed that HEV sero-prevalence in pigs was significantly associated with their production system (chi squared = 22.04, p < 0.001), with free-range pigs having much higher prevalence (91%) than confined pigs (48%). Of the 143 pigs tested in the Upper East region, 88% were sero-positive for HEV, 60% for taenia and 8% for trichinella. Overall sero-prevalence of HEV in pigs was 85%. There was no significant difference in HEV sero-prevalence between Accra and the Upper East.

## Discussion

Results indicate negligible transmission of taenia and trichinella in humans in both urban and rural settings despite poor sanitation and evidence of transmissions in pigs. Detection of Taenia and Trichinella in pigs in the Upper East region only was not surprising given the poorer sanitation and wider distribution of free-roaming pigs. A previous study in the upper East found 12% taenia cysts in pigs and no trichinella larvae [15]. The absence of these diseases in human samples indicates very low consumption of undercooked pork. Bimi et al found that 18.8% human stool samples contained taenia eggs in the nearby Northern Region. This difference may reflect different pork consumption habits in the two study areas or it might be due to the difference in diagnostic methods used. Transmission of taenia in Accra was not apparent despite regular introduction of infected pigs from Northern Ghana, most likely due to effective self-regulation of actors in the value-chain. The widespread awareness that pigs with cysts should not be consumed has resulted in an effective informal meat-inspection and condemnation procedure despite the lack of compensation. If a carcass is found to contain cysts, it is buried with quicklime on-site and the buyer selects another pig. The profits in the pig trade seem to be high enoug for pig-traders to bear these losses. The limited number of pig slaughterhouses is an advantage as good practices in any one slaughterhouse have widespread impact.

Results show high overall HEV sero-prevalence of 37% and 85% in humans and pigs respectively. This is the first study to investigate HEV simultaneously in pigs and in various groups of people in Ghana. All other studies have been on a single specific group of people such as pig-farm workers, blood donors or pregnant women [16–22].

Results showed that in Accra, men have almost two and a half times higher risk of testing positive for HEV than women for two reasons: firstly, men are better represented higher up the value chain (farmers, butchers) where the risk of zoonotic transmission is higher. Secondly, roughly 50% of cooked pork is sold and consumed at bars where the majority of the customers are men. Thus men tend to be more likely to consume undercooked pork than women. Although there was no significant difference in sex, 7 of the 10 people infected with taenia were men.

HEV correlation with occupation in humans indicates zoonotic transmission in Accra. There was no such association in the Upper East where HEV is more widespread. This may be because transmission in the Upper East is via faecally contaminated water. The relatively small number of specialist pig handlers compared to Accra might also be responsible–most people here have more general contact with pigs. Higher HEV rates in the Upper East may also be due either to the more widespread contact with pigs, or to transmission by contaminated water linked to the poorer sanitation levels. Previous studies on HEV in Ghana also indicate high sero-prevalence in at-risk groups such as pregnant women (29%), people living with HIV (45%) and pig-handlers (38%) (Adjei et al., 2009b, Feldt et al., 2013, Adjei et al., 2009a). The sero-prevalence in pig handlers in Accra (37%) matched the 38% found by Adjei et al., 2009a in the same location. Given the high rates of mortality, miscarriage and stillbirth associated with HEV in pregnancy, it is a cause for concern that 31% women of child-bearing age tested sero-positive for HEV [23–25].

The literature for SSA follows the pattern observed in Ghana in that the majority of studies are on specific high risk populations and mostly report sero-prevalences. Seropravelences between 1–80% were reported in pigs from Nigeria, Burkina Faso and Madagascar [26–29] while seroprevalences between 12% - 76% were reported in pig handlers from Uganda and Burkina Faso[30–32]. Only one study from South Africa was conducted on the general population, reporting 27.9% sero-prevalence [33]

Heavy faecal contamination of water and the environment is common in both study areas. Accra has an open defaecation rate of 45% and faecal coliform levels in drinking water of up to 1,000mpn/100ml in Kajaanor and Shiabu [34]. The Upper East region has the highest open defaecation rates in Ghana (89%) and faecal coliform levels in drinking water of up to 1,600mpn/ml in Navrongo and Bolgatanga [34, 35]. More research is required to determine which is the main route of transmission of HEV in these settings.

## Conclusions

This study shows high Hepatitis E seroprevalence in people and pigs and indicates significant transmission of taenia and trichinella in pigs in the Upper East region. Public awareness of HEV is quite low, with limited capacity for diagnosis by the health services. Confining pigs significantly reduces exposure to hepatitis E [1]. However, the absence of a legal framework to regulate pig-keeping in Accra makes it very difficult for veterinary or environmental services to engage with pig farmers in a meaningful way. We recommend that local government in Accra expands the scope of urban agriculture to include livestock and specifically, pigs. This will facilitate improved planning provision, infrastructure and regulation for urban livestock farmers and butchers. Butchers in particular are at high risk of contracting a variety of zoonotic diseases including Crimean-Congo Haemorrhagic Fever Virus, tuberculosis and brucellosis [9, 36] and more efforts should be made to educate them on these risks and on preventive measures at slaughterhouses. We present the following recommendations to fill the numerous gaps in our knowledge of HEV transmissions and burden in Ghana.

So far only seroprevalence levels are available for HEV, which indicate exposure, rather than active infection. We do not know how the incidence or true prevalence of active HEV infections in Ghana. Once these results become available, health services will need to know the proportion of these active infections which result in clinical cases and the levels of morbidity and morbidity, so that adequate resources can be allocated to diagnosis, treatment and control. This is especially important amongst pregnant women.

Secondly, what is the major route of transmission responsible or the high levels of HEV exposure seen here? Risk factors for both zoonotic and water, sanitation and hygiene (WASH) -related transmission are present. This study provides evidence *indicating* zoonotic transmission, but it has not been proved.

To address these gaps, we recommend further studies to determine true prevalence, mortality and morbidity rates as well as the HEV virus genotypes present. Only when the true extent of the problem is known can evidence based interventions be implemented. In the meantime, this and previous studies do indicate that HEV is a problem and we would recommend HEV testing by ELISA or RDT at ante-natal clinics for communities/individuals at high risk. Contact with pigs and involvement in the pork value chain have been identified as risk factors in this study. Further studies on the risk of transmission of HEV via contaminated water are also required where pigs are commonly kept or traded.

## Supporting information

S1 Appendix This is the dataset for the manuscript. (XLSX)

S2 Appendix This is the ethical approval for the study. (PDF)

S3 Appendix This is the Consent form the study. (PDF)

S4 Appendix This is the key informant interview guide for the study. (PDF)

S5 Appendix This is the focus group discussion guide for the study. (PDF)
