# Supplementary material for: Integrated value-chain and risk assessment of Pig-Related Zoonoses in Ghana
Source: PLoS One. 2019 Nov 11;14(11):e0224918. doi: 10.1371/journal.pone.0224918 (PMC6844477; doi:10.1371/journal.pone.0224918)
Supplement: S2 Appendix — (PDF) [file pone.0224918.s002.pdf]

UNIVERSITY OF GHANA
ETHICS COMMITTEE FOR BASIC AND APPLIED SCIENCES (ECBAS

P. O. Box LG 1195, Legon, Accra, Ghana

Ref. No: ECBAS 010/17-18

22nd January, 2018

Miss Vanessa Louise Magnusen
Dept. of Animal Biology and Conservation Science
University of Ghana
Legon, Accra

Dear Miss Magnusen,

ECBAS 010/17-18: IMPLEMENTATION AND IMPACT OF CURRENT PREVENTION AND CONTROL MEASURES AGAINST PIG-RELATED ZOONOTIC DISEASES IN LOW-INCOME URBAN AREAS OF GHANA

This is to inform you that the above reference study has been presented to the Ethics Committee for Basic and Applied Sciences for a full board review and the following actions taken subject to the conditions and explanation provided below:

**Expiry Date:**	21/01/19
**On Agenda for:**	Initial Submission
**Date of Submission:**	12/10/2017
**ECBAS Action:**	Approved
**Reporting:**	Bi-Annual

Please accept my congratulations.

Yours sincerely,

Professor Daniel Bruce Sarpong
ECBAS Chairperson

ETHICS COMMITTEE FOR BASIC AND APPLIED SCIENCES
APPROVED
29 JAN 2018
UNIVERSITY OF GHANA

Tel: +233-277493259 Email: ekacquaah@ug.edu.gh / ethicsbas@ug.edu.gh
