# Supplementary material for: Integrated value-chain and risk assessment of Pig-Related Zoonoses in Ghana
Source: PLoS One. 2019 Nov 11;14(11):e0224918. doi: 10.1371/journal.pone.0224918 (PMC6844477; doi:10.1371/journal.pone.0224918)
Supplement: S3 Appendix — (PDF) [file pone.0224918.s003.pdf]

UNIVERSITY OF GHANA

Official Use only<br><br>Protocol number

COLLEGE OF BASIC AND APPLIED SCIENCES

Ethics Committee for Basic and Applied Sciences (ECBAS)

PROTOCOL CONSENT FORM	
Title of Study:	Implementation and Impact of current prevention and control measures against pig-related zoonotic diseases
Principal Investigator:	Dr Ayodele O. Majekodunmi
Certified Protocol Number	ECBAS-010/17-18

General Information about Research

We want to find out how common certain diseases are in pigs, and if they are being transmitted to people who work closely with pigs and pork.

We will be carrying out the field work on study from January to March 2018

We will take blood and stool samples to check if these diseases are present. We will also carry out, focus group discussions and individual questionnaires to ask questions related to your contact with pigs and pork and any related health and safety guidelines

Benefits of the study

This research will give us a better understanding of the health risks associated with pigs pork. It will also indicate how effective current health and safety guidelines are at preventing these risks, and what can be done to improve the situation.

We do not expect any risks in the course of this study. However, there is a very minor risk of injury to pigs or handlers during blood sampling if not properly restrained.

Confidentiality

Your confidentiality is absolutely assured as we follow government regulations concerning data confidentiality .. All blood and stool samples will be handled with care and will only be used for the purpose stated. All samples will be discarded after use in line with standard protocols. Only those directly involved in this research will have access to the data from participants.

Compensation

In return for your full cooperation in this study we would like to offer a free health-check, consisting of blood pressure, blood sugar and BMI tests. We will also inform you of your test results from our research, and present the overall anonymous results to the community in a stakeholder workshop at the end of the project.

Withdrawal from Study

Your participation is voluntary and you have the right to withdraw from this study at any point without any penalty whatsoever. If we know or learn of anything that could affect your participation, you will be informed at once.

Contact for Additional Information

Dr Ayodele O. Majekodunmi

Telephone number: 0578983621

e-mail address: amajekodunmi@ug.edu.gh

Henry Ofosu Addo

Telephone number: 0244539117

e-mail address: henzorder@yahoo.com

If you have any issues on your rights as a participant you can contact the address below:

Administrator, Ethics Committee for Basic and Applied Sciences

College of Basic and Applied Sciences

University of Ghana

P. O. Box LG 68

Legon – Accra

Tel: + 233 277493259

Email: ekacquaah@ug.edu.gh

"I have read or have had someone read all of the above, asked questions, received answers regarding participation in this study, and I am willing to give consent for me, my child/ward to participate in this study. I have not waived any of my rights by signing this consent form. Upon signing this consent form, I will receive a copy for my personal records."

____

Name of Volunteer

____ ______________________

Signature or mark of volunteer Date

If volunteers cannot read the form themselves, a witness must sign here:

I was present while the benefits, risks and procedures were read to the volunteer. All questions were answered and the volunteer has agreed to take part in the research.

____

Name of witness

____ ______________________

Signature of witness Date

I certify that the nature and purpose, the potential benefits, and possible risks associated with participating in this research have been explained to the above individual.

____

Name of Person who obtained Consent

____ _____________________

Signature of Person who obtained Consent Date
