# Supplementary material for: Integrated value-chain and risk assessment of Pig-Related Zoonoses in Ghana
Source: PLoS One. 2019 Nov 11;14(11):e0224918. doi: 10.1371/journal.pone.0224918 (PMC6844477; doi:10.1371/journal.pone.0224918)
Supplement: S4 Appendix — (PDF) [file pone.0224918.s004.pdf]

Key Informant Interview Guide

Thank you for agreeing to an interview today.

My name is {namand we are conducting an assessment on Urban livestock in Accra, particularly pigs and pork on behalf of the University of Ghana, Legon. Our project is trying to understand more about urban pig value chains and their effects on veterinary and public health, food safety and the environment. The goal of this interview is to determine what exposure pathways put humans at most risk of contracting pig-related zoonotic. As a leader in this city your views and experience are extremely valuable to us and we appreciate your contribution to this research. In the interview we are most interested to gather information about pig and pork production in the city, as well as hear your views and concerns. The results of the research will be used to help decide what policies and programs related to sanitation are important to this city.

Your participation today is entirely voluntary. I would like to record notes during our interview. The reason for this is so that we do not miss anything that you say, and so we can capture your views correctly. The notes will be kept confidential and only used for this research project to improve urban agriculture. ***Is it okay to take notes during our meeting today?*** *(Confirm consent).*

The interview will last about 1 hour. Do you have any questions before we begin?

Opening Questions

1. Please provide these details

Name	
Age	
Phone number	

Email address	

2. How long have you lived this location __________?

3. What is your role as a city official here ________? Please list any specific responsibilities.

4. What is the wider role of your department in ________? Please list specific responsibilities.

5. Where is this documented? Can we access these documents?

Urban Agriculture

1. What forms of urban agriculture are practiced here?

Market garden	
Livestock	
Pigs	
Livestock markets	
others	

2. Do you/your department/officers know all pig farms in this area?

3. What are the benefits of urban agriculture?

4. What are the challenges for those practicing urban agriculture?

5. Is it sanctioned? Legal? Approved?

6. What provision is made for this under urban planning?

7. What are the regulations, laws, by-laws and policies governing urban livestock?

8. Where is this documented? Can we access these documents?

9. How are each of these policies implemented?

10. What are the practical considerations? How do they interact with customary laws and cultural practices?

11. How do farmers dispose of animal dung?

12. Where do they get water for their animals/gardens?

13. How many veterinary/environmental/planning officers are in this area?

14. Do they know the locations of all pig farms in this area?

15. Do they visit/inspect them? How often?

16. What do they look out for when they visit/inspect pig farms?

Animal housing	
feed	
cleanliness	
others	

17. What actions do they take against offenders?

18. What effects do you feel urban livestock have on

Environment	
Veterinary health	
Public health	

19. Is there any documented evidence to your suggestions?

Pig Health

1. What are the regulations, laws, by-laws and policies governing veterinary public health in urban areas? *(animal health, zoonotic disease, meat inspection, food safety at slaughter slabs and food vendors, transportation of live animals and raw meat, livestock markets)*

2. How do they differ from those in peri-urban and rural areas?

3. Where is this documented? Can we access these documents?

4. How many veterinarians/paraveterinarians are there in the department? Is this number adequate?

5. How are each of these policies implemented? What are the procedures for ensuring veterinary public health? (inspection, vaccination, extension and training for farmers, routine treatments, on demand treatments?)

6. What diseases are covered? (notifiable? Common? Severe? Zoonotic?)

7. What are the practical considerations? How do they interact with customary laws and cultural practices?

8. What is the interaction with other departments/officials? (public health, environmental, etc). Who is responsible for what?

9. Do you ever get requests for meat inspection? Who from? In what circumstances?

10. What are the procedures for reporting disease or other incidents? Are they well-used? Where is this recorded/documented? What action is taken?

WASH & Environmental Issues

20. What are the main water sources in the district? Drinking water? Bathing/household water?

21. Where do farmers get water for their livestock? Farms?

22. Do you have an estimate of sanitation facilities in the area?

23. How many public toilets are there in the area? Is it common for people in this city to use public latrines?

24. How many compounds/households have their own toilets?

25.

26. What other methods are used for solid waste disposal? Is it common for people in this city to shit in the open?

27. What are the regulations, laws, by-laws and policies governing domestic water and sanitation issues?

28. Where is this documented? Can we access these documents?

29. How are each of these implemented?

30. What are the practical considerations? How do they interact with customary laws and cultural practices?

1. How many environmental/public health officers are in this area?

2. Do they visit/inspect households and public areas? How often?

3. What do they look out for when they visit/inspect?

4. What actions do they take against offenders?

31. In what ways do you think people in your district come into contact with fecal matter? What provision is made for this under urban planning?

5. Are there any oceans, rivers, lagoons, or ponds in this city? If so, please provide the name and the neighborhood they are located in or near. (Take map along!)

6. Do you know if sewage is disposed of directly into any of the bodies of water or drains in the city? If so, where and how?

7. What rubbish/waste disposal facilities are available for households?

8. Is there a functional water treatment plant in this district?

32. How do you generally feel about the city's water quality? Probe: Is there any documented evidence to your suggestions?

33. How do you generally feel about the way waste/sewage is managed in the city?

34. To the best of your knowledge, are any of the fruits or vegetables produced and /or consumed in the city irrigated with wastewater or biosolids?

35. Do you think there is fecal contamination in the piped water supply?

36. If and when it floods, is it likely that there is fecal contamination in the floodwater?

37. Are there any particular parts of this city or specific communities that are known to be contaminated or have very poor sanitation? Think about any bodies of water, large venues, or facilities, name the communities in order of poor sanitation?

1. What effects do you feel water and sanitation issues have on the

Environment	
Veterinary health	
Public health	

38. Is there any documented evidence to your suggestions?

Livestock Slaughter and raw meat sale

1. Are there any slaughter houses in the district?

a	Name	
b	Location	
c	Animals slaughtered	
d	Size/number of animals per day?	

2. Do you/your department/officers know all slaughter sites in this area?

3. Are they sanctioned? Legal? Approved?

4. What provision is made for this under urban planning?

5. Where do they get water for their operations?

6. How do they dispose of their waste: Blood, bone, offal, waste water?

7. How is the raw meat transported and sold after slaughter? Are there specific markets for raw meat? Where are these sales points?

8. What about home/private/individual slaughter

9. What are the regulations, laws, by-laws and policies governing livestock slaughter and the sale of raw meat?

10. Where is this documented? Can we access these documents?

11. How are each of these policies implemented?

12. What are the practical considerations? How do they interact with customary laws and cultural practices?

13. Are these slaughter houses and sales points inspected by environmental, veterinary, planning, public health officers? How often?

14. What do they look out for when they visit/inspect slaughter houses and raw meat vendors?

15. What actions do they take against offenders?

16. What effects do you feel urban livestock have on the

Environment	
Veterinary health	

Public health	

1. What are the benefits of these pork vendors to the community?

2. What are the challenges for the vendors?

3. Is there any documented evidence to your suggestions?

Food vendors

4. Are there any pork vendors in the district?

a	Name	
b	Location	
c	Animals slaughtered	

d	Size/number of animals per day?	

5. Do you/your department/officers know all pork vendors in this area?

6. Are they sanctioned? Legal? Approved?

7. What provision is made for this under urban planning?

8. Where do they get water for their operations?

9. How do they dispose of their waste materials and waste water?

10. How the pork is generally processed and sold?

11. What are the regulations, laws, by-laws and policies governing food vendors in this district?

12. Where is this documented? Can we access these documents?

13. How are each of these policies implemented?

14. What are the practical considerations? How do they interact with customary laws and cultural practices?

15. Are pork vendors inspected by environmental, veterinary, planning, public health officers?

    How often?

16. What do they look out for when they visit/inspect food vendors?

17. What actions do they take against offenders?

18. What effects do you feel urban livestock have on:

Environment	
Veterinary health	
Public health	

19. What are the benefits of these pork vendors to the community?

20. What are the challenges for the vendors?

21. Is there any documented evidence to your suggestions?

Closing Questions

39. What do you think are the biggest environmental health or water and sanitation related problems in the city related to infrastructure, policy, and behavior?

40. Why do you think these problems are an issue here?

41. Is there a particular part of the district where you think these problems may be the worst? If so, Why? (Probe for specific areas/neighborhoods)

42. For whom (what subsections of the population) do you think these problems are the worst? Why?

43. Is there any further information or data that you are able to provide? (Do you have maps of the neighborhood or water source locations or public latrines? Do you have estimates of facility usage?)

