# Supplementary material for: Integrated value-chain and risk assessment of Pig-Related Zoonoses in Ghana
Source: PLoS One. 2019 Nov 11;14(11):e0224918. doi: 10.1371/journal.pone.0224918 (PMC6844477; doi:10.1371/journal.pone.0224918)
Supplement: S5 Appendix — (PDF) [file pone.0224918.s005.pdf]

Value Chain Focus Group Discussion

Interviewer Name ________________ Date ________ Location ________________.

Dear Participant,

This questionnaire is based on the study of the Risk of Parasitic Zoonoses along the Pork Value Chain in Accra. The study is carried out by University of Ghana students and staff and seeks better understanding of the pig and pork industry in Accra.

We will ask you a few questions about yourself, your business and the locations involved. We would be glad if you could spend a few minutes of your time to answer the questions as sincerely as you can.

Responses will be used for the intended study only. We ensure confidentiality of your responses and personal information. Participation in this survey is completely voluntary and participants may withdraw at any time without any penalty. Also, participants will not be adversely affected if they decline to participate or later stop participating.

"I have read or have had someone read all of the above, and I am willing to give consent to participate in this study. I have not waived any of my rights by signing this consent form. Upon signing this consent form, I will receive a copy for my personal records."

____ ________

Signature or Mark of Volunteer Date

SECTION A - Demographic Information

Name	Gender	Age	Level of Education
1.			
2.			
3.			
4.			
5.			
6.			
7.			
8.			
9.			
10.			
11.			
12.			

SECTION B –Locations

4. Where do you live?

5. Where do you buy your pigs?

6. Where do the pigs come from?

7. Why do you choose to buy your pigs there?

8. How are the pigs transported here?

9. How do you select your pigs? What do you look out for?

10. Do you ever get requests for home slaughter? Who from? In what circumstances?*

11. Please list locations where you have previously carried out home slaughter*[i]

SECTION C – Your Business

12. Why have you chosen this trade rather than any other business/profession *(experience in the business, profitable business, high demand)*

13. Is this your main source of livelihood? Or supplementary income?

14. Why do you choose to do your butchering work here?*

15. How and where did you train for this job?*

16. How many pigs do you buy every month? At what price?

17. Do prices remain constant or fluctuate over time? Why?

(T*ime of year? Source of pig? Feed prices? sellers whim?*)

18. How much bargaining power do you have?

19. How many pigs do you sell/slaughter every month? At what price?

20. What determines the price?

21. Do prices remain constant or fluctuate over time? Why

(T*ime of year? Source of pig? Feed prices? sellers whim?*)

22. Who are your customers? Where are they from? What do they do with your products?

23. Apart from the cost of the pig, what other production costs do you have for your business? Please rank in order of magnitude

(Pig feed; gas/charcoal/firewood; knives, etc; water; ropes; electricity; permits; transport; water; other)

24. What are the constraints to your business?

25. When did you start this business? Has your business grown since then? How?

(*No. of pigs sold or slaughtered per month at the start compared to now, number of staff, volume of customers, additional items sold, etc)*

26. What are the barriers to expanding your business?

SECTION C - Linkages

27. Do you have any interactions with environmental officers? Describe their activities?

28. How do you rate the impact of their work?

(Good/bad? Helpful/Obstructive? High/Low impact?)

29. Any other authorities?

(formal authorities – vets, town council, etc; informal authorities – market queens, associations, etc)

30. Do you have links to those in the same business with you? What links?

31. Is this current state of linkages good enough?

32. What are your suggestions for improvement of within stakeholder group?

33. Do you have links to other stakeholders in the pig/pork value chain?

*(Links **apart from personal or customer-supplier relationships**, e.g. associations, regular meetings, sources of information, etc; **Other stakeholders such as** farmers, butchers, meat sellers, food sellers, cold stores, etc)*

34. Is this current state of linkages good enough?

35. What are your suggestions for improvement of linkages within the stakeholder group

36. What are your suggestions for improvement of linkages between stakeholder groups?

SECTION D – Zoonoses

37. Do you inspect live pigs for signs of disease? What do you look out for?

38. Do you inspect carcasses for signs of disease? What do you look out for?

39. Are you aware of diseases in animals/meat that can affect human beings?

40. If yes, which ones?

41. If mentioned by respondent, what do you know about tape worms?

42. Do you ever see cysts in meat? What do you know about them?

(Where do they come from? What is inside them? Why are they there? Can they be eaten?)

43. Where present what do you do about them?

1. Do you ever request meat inspection? Who from? In what circumstances?

2. Do you ever get requests for home slaughter? Who from? In what circumstances?*
3. Please list locations where you have previously carried out home slaughter*
4. What are the procedures for reporting disease or other incidents? Are they well-used? What action is taken?

Section E – Water and sanitation

What is your main source of water?

5. What toilet facilities do you have here?

6. Do you know of any regulations, laws, governing water and sanitation issues?

7. How are these implemented/enforced?

8. What actions is taken against offenders?

Section F – Interactions with authorities

1. Are these premises approved by the district urban planning department? Before or after the business started?

2. Does the urban planning department make any provisions for your activities??

3. Are these premises inspected by environmental, veterinary, planning, public health officers? How often?

4. What do they look out for when they visit?

5. Are you aware of any regulations, laws, by-laws and policies governing your work?

(animal health, zoonotic disease, meat inspection, food safety, transportation of live animals and raw meat, livestock markets)

1. How are these policies implemented/enforced?

6. What actions is taken against offenders?

7. Do they inspect live pigs or carcasses for signs of disease? What do they look out for?

8. What effects do you feel your activities have on the environment? Animal Health? Public Health?

[i] *Butchers only
